# High Resolution Haplotype Analyses of Classical HLA Genes in Families With Multiple Sclerosis Highlights the Role of HLA-DP Alleles in Disease Susceptibility

**DOI:** 10.3389/fimmu.2021.644838

**Published:** 2021-05-25

**Authors:** Kazutoyo Osoegawa, Lisa E. Creary, Gonzalo Montero-Martín, Kalyan C. Mallempati, Sridevi Gangavarapu, Stacy J. Caillier, Adam Santaniello, Noriko Isobe, Jill A. Hollenbach, Stephen L. Hauser, Jorge R. Oksenberg, Marcelo A. Fernández-Viňa

**Affiliations:** ^1^ Histocompatibility & Immunogenetics Laboratory, Stanford Blood Center, Palo Alto, CA, United States; ^2^ Department of Pathology, Stanford University School of Medicine, Palo Alto, CA, United States; ^3^ Weill Institute for Neurosciences, Department of Neurology, University of California San Francisco, San Francisco, CA, United States; ^4^ Department of Neurology, Graduate School of Medical Sciences, Kyushu University, Fukuoka, Japan

**Keywords:** multiple sclerosis (MS), family, HLA, haplotype, transmission disequilibrium test (TDT), case-control analysis

## Abstract

Multiple sclerosis (MS) susceptibility shows strong genetic associations with HLA alleles and haplotypes. We genotyped 11 HLA genes in 477 non-Hispanic European MS patients and their 954 unaffected parents using a validated next-generation sequencing (NGS) methodology. HLA haplotypes were assigned unequivocally by tracing HLA allele transmissions. We explored HLA haplotype/allele associations with MS using the genotypic transmission disequilibrium test (gTDT) and multiallelic TDT (mTDT). We also conducted a case-control (CC) study with all patients and 2029 healthy unrelated ethnically matched controls. We performed separate analyses of 54 extended multi-case families by reviewing transmission of haplotype blocks. The haplotype fragment including DRB5*01:01:01~DRB1*15:01:01:01 was significantly associated with predisposition (gTDT: *p* < 2.20e-16; mTDT: *p* =1.61e-07; CC: *p* < 2.22e-16) as reported previously. A second risk allele, DPB1*104:01 (gTDT: *p* = 3.69e-03; mTDT: *p* = 2.99e-03; CC: *p* = 1.00e-02), independent from the haplotype bearing DRB1*15:01 was newly identified. The allele DRB1*01:01:01 showed significant protection (gTDT: *p* = 8.68e-06; mTDT: *p* = 4.50e-03; CC: *p* = 1.96e-06). Two *DQB1* alleles, DQB1*03:01 (gTDT: *p* = 2.86e-03; mTDT: *p* = 5.56e-02; CC: *p* = 4.08e-05) and DQB1*03:03 (gTDT: *p* = 1.17e-02; mTDT: *p* = 1.16e-02; CC: *p* = 1.21e-02), defined at two-field level also showed protective effects. The HLA class I block, A*02:01:01:01~C*03:04:01:01~B*40:01:02 (gTDT: *p* = 5.86e-03; mTDT: *p* = 3.65e-02; CC: *p* = 9.69e-03) and the alleles B*27:05 (gTDT: *p* = 6.28e-04; mTDT: *p* = 2.15e-03; CC: *p* = 1.47e-02) and B*38:01 (gTDT: *p* = 3.20e-03; mTDT: *p* = 6.14e-03; CC: *p* = 1.70e-02) showed moderately protective effects independently from each other and from the class II associated factors. By comparing statistical significance of 11 HLA loci and 19 haplotype segments with both untruncated and two-field allele names, we precisely mapped MS candidate alleles/haplotypes while eliminating false signals resulting from ‘hitchhiking’ alleles. We assessed genetic burden for the HLA allele/haplotype identified in this study. This family-based study including the highest-resolution of HLA alleles proved to be powerful and efficient for precise identification of HLA genotypes associated with both, susceptibility and protection to development of MS.

## Introduction

Multiple sclerosis (MS) is a chronic debilitating neurological disorder associated with central nervous system inflammation, demyelination, and axonal degeneration (1). MS is considered a multifactorial disease with abundant evidence linking multiple common alleles across the genome to disease risk and progression (2, 3). The most notable susceptibility genomic region for MS maps to the Major Histocompatibility Complex (MHC) in chromosome 6p21.3 (4–10), where distinct HLA alleles have been consistently found associated with both, susceptibility and protection.

In addition to bidirectional influences on risk, HLA allelic and haplotypic heterogeneity has been reported (11), together with population effects (12), epistasis (13), and sex dimorphism (14, 15), reflecting the biological complexity of the mechanisms underlying the statistical associations. Different study designs and the evolution of genotyping methods also contributed to the identification of primary and secondary associations, enriching the working models that describe the role of HLA gene products in disease predisposition.

The recent advent of cost-effective next generation sequencing (NGS) methods and customized algorithms allow to sequence nearly complete HLA genes including UTRs and introns, to uncover the nucleotide variation on study groups including novel variants, and to assign the least ambiguous HLA genotypes and untruncated haplotypes (16).

As part of the disease association and family haplotype projects for the 17^th^ International HLA and Immunogenetics Workshop (IHIW), we generated high-resolution data for 11 HLA genes in 477 MS trio families. We took advantage of recently developed algorithms to track the segregation of NGS HLA genotypes and performed a classical transmission disequilibrium test (TDT) analysis (17) to further improve or understanding of the HLA allelic and haplotypic disease-associated landscape.

## Materials and Methods

### Study Subjects

In total 477 trio MS families, totaling 1431 subjects with non-Hispanic European ancestry are included. A trio includes one proband diagnosed with MS and two unaffected parents. All children met established diagnostic criteria of MS (18). Transmission and non-transmission of parental alleles and haplotypes was evaluated to assess associations with MS. In addition, associations were examined in a case-control analysis by comparing the distribution of HLA alleles in these 477 children with those in a control group including 2029 previously characterized unrelated healthy subjects with non-Hispanic European ancestry as control group (19).

There were 54 additional families with multiple MS cases (n = 147) found in multiple generations that included samples with European ancestry. **Table 1** shows clinical demographic information for the MS cases for trio and extended families. We did not perform disease association statistical analyses for the extended families but reviewed the HLA allele/haplotype inheritance patterns within the families. **Supplemental Table 1** includes summary of the extended families.

**Table 1:** Clinical and demographic information in MS patient from trios and extended families.

Disease course	Trios	Extended families
	Cases (N=477)	Cases (N=147)
Clinically Isolated Syndrome (CIS)	0	4
Relapse-Remitting (RR)	382	82
Secondary Progressive (SP)	90	29
Primary Progressive (PP)	1	5
Progressive-Relapsing (PR)	1	3
Unknown sub-type	3	244
		
**Gender**		
Female	373	104
Male	104	43
		
**Age of disease onset**		
Range	11- 50	12- 54
Mean	28.9	30.5
Median	28	29.5

Table contains “Disease course”, “Gender” and “Age of disease onset” information. “Trio” column shows demographic information about MS cases (children) from 477 trio families. “Extended families” column shows demographic information about MS cases from 54 extended families in which the numbers of MS patients ranged from 2 to 7.

This study was approved by the University of California, San Francisco Institutional Review Board. The analyses of HLA genotype data with the double-blinded sample IDs were conducted at the Stanford Blood Center and Stanford University under the Stanford University Institutional Review Board (IRB) eProtocol titled, “17th International HLA and Immunogenetics Workshop” (#: 38899).

#### Genotyping

We initially processed DNA from the above subjects for typing all alleles at the 11 classical HLA loci (*HLA-A*, *-C*, *-B*, *-DRB3* ,]*-DRB4*, *-DRB5*, *-DRB1*, *-DQA1*, *-DQB1*, *-DPA1*, and *-DPB1*) using MIA FORA NGS FLEX HLA Typing Kits (Immucor Inc.) (20), and sequenced using NextSeq and MiniSeq DNA sequencers (Ilumina Inc.). We used MIA FORA 3.1 software with IPD-IMGT/HLA Database release version 3.25.0 for DNA sequence assembly and HLA genotype assignments. HLA genotypes were extracted in Histoimmunogenetics Markup Language (HML) format (21) and imported into 17^th^ International HLA and Immunogenetics Workshop (17^th^ IHIW) database (22). Some individuals in the extended families had no DNA, and missing HLA genotypes were manually imputed previously described (23). The allelic and genotypic (or “phase”) ambiguities were present in HLA genotypes due to the technical and methodological limitations, and reported previously (24). We included the ambiguities for the haplotype analyses (24, 25), but reported only the lowest-digit allele name in this manuscript, e.g. HLA-DRB1*15:01:01:01. For genotypic ambiguities (e.g. HLA-DPB1*04:01:01:01+HLA-DPB1*04:02:01:02|HLA-DPB1*105:01+HLA-DPB1*126:01), it is possible to identify a single phased allele combination in most of the instances by reviewing all the genotypes in the family and assessing segregation (24). When the genotypic ambiguity is not resolved due to the lack of informative family members, the lowest-digit allele name combination is used as the priority HLA genotype (24). HLA genotyping data for the 477 trio families was submitted to the Immport data warehouse (https://www.niaid.nih.gov/research/immport).

### Building HLA Haplotypes

The family ID, subject ID, familial relationship and HLA genotypes organized in Genotype List String (GL string) format (26) were downloaded from the 17^th^ IHIW database (22) and used as input format to build HLA haplotypes from trios using HaplObserve software (24). HaplObserve generates an output file containing “transmitted” and “non-transmitted” haplotypes/alleles counts in comma separated value (CSV) file that allows keeping track of which haplotypes were transmitted or not transmitted to the offspring. The haplotypes were separated into the small haplotypes/alleles as described previously (24). **Supplemental Table 2** contains “transmitted” and “non-transmitted” haplotype counts for 11-loci, Class I, *HLA-DRB3/4/5*~*HLA-DRB1*~*HLA-DQA1*~*HLA-DQB1*, *HLA-DPA1*~*HLA-DPB1* haplotypes with both untruncated and two-field allele determinations.

### Genotypic Transmission Disequilibrium Test (gTDT) and Multiallelic TDT (mTDT)

The HLA genotypes represent a combination of HLA alleles or HLA haplotypes for target HLA loci.

First, we performed TDT with HLA genotypes from trio families using “Genotypic TDT “ function in trio version 3.18.0 R package (27–29). We use gTDT as an abbreviation for Genotypic TDT in this manuscript. We tested both untruncated (e.g. HLA-A*02:01:01:01) and two-field (e.g. HLA-A*02:01) allele names for 11 HLA genes (see section *Genotyping*), and 19 HLA haplotype blocks (*HLA-A*~*HLA-C*~*HLA-B*, *HLA-A*~*HLA-C*~*HLA-B*~*HLA-DRB1*~*HLA-DQB1*, *HLA-A*~*HLA-C*~*HLA-B*~*HLA-DRB3/4/5* ~*HLA-DRB1*~*HLA-DQA1*~*HLA-DQB1*, *HLA-B*~*HLA-DQB1*, *HLA-B*~*HLA-DRB1*, *HLA-C*~*HLA-B*, *HLA-C*~*HLA-B~HLA-DRB1*~*HLA-DQB1*, *HLA-C*~*HLA-B*~*HLA-DRB3/4/5*~*HLA-DRB1*~*HLA-DQA1*~*HLA-DQB1*, *HLA-C*~*HLA-B*~*HLA-DRB3/4/5* ~*HLA-DRB1*~*HLA-DQA1*~*HLA-DQB1~HLA-DPA1*~*HLA-DPB1*, *HLA-C*~*HLA-DQB1*, *HLA-C*~*HLA-DRB1*, *HLA-DPA1*~*HLA-DPB1*, *HLA-DQA1*~*HLA-DQB1*, *HLA-DRB3/4/5*~*HLA-DRB1*, *HLA-DRB1*~*HLA-DQB1*, *HLA-DRB1*~*HLA-DQB1~HLA-DPB1*, *HLA-DRB3/4/5*~*HLA-DRB1*~*HLA-DQA1*~*HLA-DQB1*, *HLA-DRB3/4/5*~*HLA-DRB1*~*HLA-DQA1*~*HLA-DQB1~HLA-DPA1*~*HLA-DPB1*, *HLA-A*~*HLA-C*~*HLA-B*~*HLA-DRB1*~*HLA-DQB1*, *HLA-A*~*HLA-C*~*HLA-B*~*HLA-DRB3/4/5*~*HLA-DRB1*~*HLA-DQA1*~*HLA-DQB1~HLA-DPA1*~*HLA-DPB1*). We chose these blocks to eliminate false signals resulting from ‘hitchhiking’ alleles.

In addition, we performed a second TDT analysis with HLA genotypes from the trio families using the Transmission/disequilibrium test of a multiallelic marker by Bradley-Terry model (mtdt2) function in gap version 1.2.2 R package (30–32). We use mTDT as an abbreviation for multiallelic marker TDT. Similar to TDT, we tested both untruncated and two-field allele names for 11 HLA genes, and 19 HLA haplotype blocks as described above.

We performed various stratification gTDT and mTDT analyses, which excluded families and individuals that carried target allele/haplotype. For example, to eliminate the risk effect of haplotype bearing HLA-DRB1*15:01, we removed all the families that contained at least one family member with the risk haplotype bearing HLA-DRB1*15:01 in the data set. Finally, we performed conditional logistic regression TDT analyses using colGxE function in trio R package in the presence or absence of the haplotype bearing HLA-DRB1*15:01 to determine the effect and its interaction with the other HLA alleles/haplotypes (29, 33). To identify the genetic model underlying the association between HLA haplotypes/alleles and MS, we also performed the MAX gTDT to compute the maximum over the TDT statistics for an additive, dominant, and recessive model, and to compute permutation-based p-values (28).

### Case-Control (CC)

For case-control analyses, we used Bridging ImmunoGenomic Data-Analysis Workflow Gaps (BIGDAWG) (34). We tested the same 11 HLA genes, and 19 haplotypes for CC analyses using BIGDAWG. Similar to gTDT and mTDT, we also performed various stratification CC analyses.

### Summarizing gTDT, mTDT, and CC Results

Sixty [(11 loci + 19 haplotypes) x 2 (untruncated and two-field)] different tests were performed for gTDT, mTDT and CC. Each software package for gTDT, mTDT and CC generates different format of output files. To circumvent manual manipulation of the output files, and capture essential information from the output files, we developed a script to summarize the results in a single file using Practical Extraction and Report Language (Perl) programming. We compared gTDT, mTDT and CC results for all observed alleles and haplotypes (**Supplemental Tables 3**–**12**). We used a threshold of significance of 0.05 for gTDT, mTDT and CC.

### Deviations From Expected Hardy-Weinberg Equilibrium (HWE)

Python for Population Genomics (PyPop) version 0.7.0 (35) was used to investigate Hardy-Weinberg Equilibrium (HWE) *via* the Guo and Thompson test (36), assessing genotyping proportions for both individual loci. We identified individual genotypes deviating significantly from HWE expectations using Chen’s method (37), using a threshold of significance of 0.05.

### Measuring MS Risk-Protective Effects

We assessed the risk-protective effects of the identified risk and protective HLA alleles/haplotypes in two approaches. First, we recorded the presence of a risk allele/haplotype to be +1 and a protective allele/haplotype to be -1, and calculated the sum of the scores for each case-control study subject. We classified each subject into five categories for both cases and controls based on the final net scores: 1) Risk (positive score); 2) Neutral Zero (no risk and protective factor present); 3) Neutral Risk Protective (equivalent numbers of risk and protective factors); 4) Protective Risk (negative score, but at least one copy of risk allele present); 5) Protective (protective allele only). **Supplemental Table 13A** shows the summary for each category. We calculated 2 x 2 odds ratio for Risk *vs*. Neutral Zero, Protective *vs*. Neutral Zero and Neutral Risk Protective *vs.* Neutral Zero to measure the risk and protective effects (**Supplemental Tables 13B–D**) (38).

Second, we calculated HLA genetic burden (HLAGB) for each study subject as the sum of the burden of each MS associated HLA allele as described (39, 40). Each allele burden was calculated as the allele dose multiplied by the allele effect size obtained in this study. We generated box plots for HLAGB scores using “ggplot2” package in R programming language.

### DNA Sequence Alignment

We downloaded genomic DNA sequences of HLA-DPA1*01:03:01:02 (HLA06604) and HLA-DPB1*104:01:01:01 (HLA02046) from IPD-IMGT/HLA Database release version 3.35.0 (41), and compared them with HLA-DPA1*01:03:01:03~HLA-DPB1*03:01:01 haplotype (Accession Number: AL662824) (42), because we did not sequence 5’-UTR, exon 1 and intron 1 of *HLA-DPB1* (20). We used the BLAT DNA sequence alignment tool in the UCSC Genome Browser (43).

### Expression Quantitative Trait Loci (eQTL)

We investigated expression quantitative trait loci (eQTL) for *HLA-DPA1* and *HLA-DPB1* using GTEx Portal database (https://www.gtexportal.org/home/) (44).

## Results

### Principal Susceptibility HLA Class II Alleles and Haplotype Blocks

In agreement with previous observations, the HLA-DRB5*01:01:01~HLA-DRB1*15:01:01:01 haplotype block was significantly associated with susceptibility to MS in this dataset (gTDT: RR = 3.42, CI = 2.65-4.42, *p* = < 2.20e-16; mTDT: *p* = 1.61e-07; CC: OR = 3.02, CI = 2.55-3.58, *p* = < 2.22e-16) (**Table 2**).

**Table 2:** Main HLA alleles and haplotype fragments associated with susceptibility and protection to MS.

Allele or Haplotype fragment	TDT			mTDT	CC			Effect
	RR	CI	*p*-value	*p*-value	OR	CI	*p*-value	
DRB5*01:01:01~DRB1*15:01:01:01	3.42	2.65-4.42	< 2.20e-16	1.61e-07	3.02	2.55-3.58	< 2.22e-16	Predisposing
DPB1*104:01	2.90	1.41-5.95	3.69e-03	2.99e-03	1.76	1.10-2.74	1.00e-02	Predisposing
DRB1*01:01:01	0.38	0.25-0.59	8.68e-06	4.50e-03	0.44	0.30-0.62	1.96e-06	Protective
DQB1*03:01	0.68	0.53-0.88	2.86e-03	5.56e-02	0.67	0.55-0.81	4.08e-05	Protective
DQB1*03:03	0.52	0.32-0.87	1.17e-02	1.16e-02	0.57	0.35-0.89	1.21e-02	Protective
DPB1*09:01:01	0.10	0.01-0.78	2.81e-02	3.22e-02	0.15	0.00-0.92	3.20e-02	Protective
A*02:01:01:01~C*03:04:01:01~B*40:01:02	0.30	0.13-0.71	5.86e-03	3.65e-02	0.40	0.16-0.82	9.69e-03	Protective
B*38:01	0.38	0.20-0.72	3.20e-03	6.14e-03	0.50	0.26-0.90	1.70e-02	Protective
B*27:05	0.38	0.22-0.66	6.28e-04	2.15e-03	0.55	0.32-0.90	1.47e-02	Protective
B*44:02	0.62	0.43-0.89	9.60e-03	8.94e-02	0.62	0.45-0.86	3.14e-02	Protective

Table shows susceptible and protective associations with MS of HLA alleles and haplotype fragments examined by TDT, mTDT and CC. RR, CI and OR represent relative risk, confidence interval, and odds ratio, respectively. The TDT and mTDT results are obtained from 477 trio families. The CC results are generated from 477 cases and 2029 controls. Allele names for DQB1*03:01, DQB1*03:03, B*38:01, B*27:05 and B*44:02 are shown as truncated two-field allele names.

Of 477 trio families, 301 families carried at least one HLA-DRB5*01:01:01~HLA-DRB1*15:01:01:01 haplotype, and 252 families had one MS-affected child with the HLA-DRB5*01:01:01~HLA-DRB1*15:01:01:01 haplotype. The HLA-DRB5*01:01:01~HLA-DRB1*15:01:01:01 extended haplotype block in this dataset includes several common class I *HLA-C~HLA-B* embedded haplotypes: e.g. HLA-C*07:02:01:03~HLA-B*07:02:01, HLA-C*07:01:01:01~HLA-B*08:01:01:01, HLA-C*12:03:01:01~HLA-B*18:01:01:02 and the minor *HLA-DQA1~HLA-DQB1* haplotype: HLA-DQA1*01:02:01:01~HLA-DQB1*06:03:01. We observed reduced significance of these extended haplotypes across the telomeric and centromeric segments of the locus (**Supplemental Table 3**) and concluded that the HLA-DRB5*01:01:01~HLA-DRB1*15:01:01:01 short block constitutes the core element of the risk haplotype. HWE testing for 477 affected children showed HLA-DRB1*03:01:01:01 homozygosity excess (11 observed; 6.4 expected; *p* = 0.0442). Upon application of conditional logistic regression TDT analyses, and consistent with previous reports (4, 5), HLA-DRB1*03:01 behaved as a recessive MS risk allele in this dataset (RR = 4.48, CI = 1.73-11.62, *p* = 2.03e-3).

**Figure 1:**
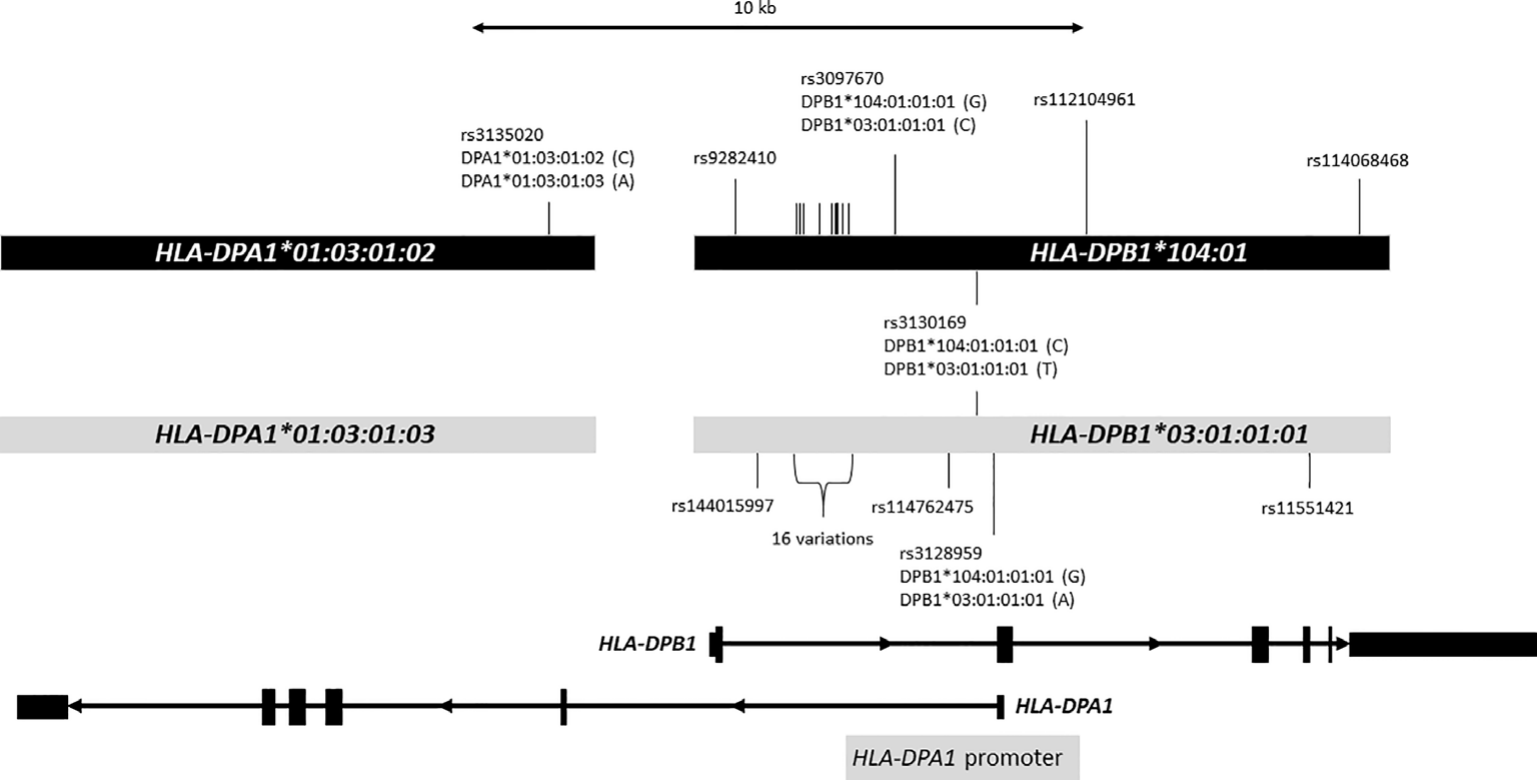
Distinct two DP haplotypes. Figure shows a 25-kb genomic region containing *HLA-DPA1* and *HLA-DPB1*. *HLA-DPA1* and *HLA-DPB1* are shown at the bottom. Exons are depicted with rectangles, and introns are shown with thin lines with arrow that indicates the direction of transcription. The light gray bars in the middle represent DNA sequences containing HLA-DPA1*01:03:01:03 and HLA-DPB1*03:01:01:01 alleles, and the black bars represent DNA sequences containing HLA-DPA1*01:03:01:02 and HLA-DPB1*104:01:01:01 alleles. The DNA sequences of HLA-DPA1*01:03:01:02 and HLA-DPB1*104:01:01:01 alleles obtained from IPD-IMGT/HLA Database release 3.35.0 were aligned to the HLA-DPA1*01:03:01:03 and HLA-DPB1*03:01:01:01 allele sequences using the BLAT DNA sequence alignment tool. We identified 26 nucleotide variations (SNPs) indicated with vertical bars between these haplotypes; only 10 SNPs are shown with *rs* numbers, SNP IDs were omitted for the remaining 16 SNPs (indicated as 16 variations: rs2213307, rs2213308, rs2213309, rs2856816, rs28449420, rs7750458, rs115244420, rs116358647, rs115735976, rs2073520, rs2073521, rs2073522, rs2073523, rs7754299, rs114364259, rs114227078). Of 26 SNPs, 22 are located in intron 1 of *HLA-DPB1*. *HLA-DPA1* promoter depicted with gray bar at the bottom is located in the complementary strand of genomic DNA sequence that overlaps with intron 1 and exon2 of *HLA-DPB1*. We observed statistically significant 4 eQTL SNPs highlighted in red. Nucleotides corresponding to *HLA-DPB1* alleles for these 4 SNPs were shown in parentheses. For instance, HLA-DPB1*104:01:01:01 and HLA-DPB1*03:01:01:01 have “G” and “A”, respectively, for rs3128959.

In addition, we observed distorted segregation of the HLA-DPB1*104:01 allele (gTDT: RR = 2.9, CI = 1.41-5.95, *p* = 3.69e-03; mTDT: *p* = 2.99e-03; CC: OR = 1.76, CI = 1.10-2.74, *p* = 1.00e-02) (**Table 2** and **Supplemental Table 4A**). Of 477 trio families, 37 families (one parent for 33 families and both parents for 4 families) carried the HLA-DPB1*104:01 allele, and 29 families had one MS-affected child with the allele HLA-DPB1*104:01. To exclude the possibility that the *HLA-DPB1* susceptibility signal may derive from the primary HLA-DRB1*15:01 association, we performed a stratification analysis, which excluded families and individuals that carried the haplotype bearing HLA-DRB1*15:01, confirming that HLA-DPB1*104:01 is independently associated with MS risk (**Supplemental Table 4B**). We confirmed the independent association of the allele HLA-DPB1*104:01 with conditional logistic regression TDT analysis (RR = 4.2, CI = 1.58-11.14, *p* = 3.93e-03). We did not observe an interaction between the allele HLA-DRB1*15:01 and the allele HLA-DPB1*104:01. Out of 41 parents carrying the HLA-DPB1*104:01 allele, 7 parents were HLA-DRB1*15:01 carriers, but all of them were present on the other copy of chromosome 6, further confirming that HLA-DPB1*104:01 risk haplotypes are independently segregated to their affected offspring. Interestingly, the closely related allele HLA-DPB1*03:01:01 did not show any significant association (**Figure 1** and **Supplemental Table 4A**).

### Protective HLA Class II Alleles and Haplotype Blocks

The allele HLA-DRB1*01:01:01 showed the strongest class II protective effect in this dataset (**Table 2** and **Supplemental Table 5A**). As shown previously, HLA-DRB1*01:01 is protective for MS in even the presence of HLA-DRB1*15:01 (4, 45). Of 252 children who carry at least one haplotype bearing HLA-DRB1*15:01, we identified 9 (3.5%) children who also carry the HLA-DRB1*01:01:01 allele. Of 370 parents who carry at least one haplotype bearing HLA-DRB1*15:01, we found 42 (11.3%) parents who also carry the HLA-DRB1*01:01:01 allele. The odds ratio confirms the statistically significant protective effect of HLA-DRB1*01:01:01 allele in the presence of haplotype bearing HLA-DRB1*15:01 (OR = 0.29; CI = 0.14-0.61; Yates *p* = 0.000892). Conditional logistic regression TDT analysis suggested a statistically significant interaction between HLA-DRB1*15:01 and HLA-DRB1*01:01 (RR = 0.17, CI = 0.08-0.38, 2 degree of freedom Wald test *p* = 1.34e-05). When eliminating families with haplotypes bearing HLA-DRB1*15:01 (**Supplemental Table 5B**), the HLA-DRB1*01:01:01 allele showed weak protective effects for gTDT, mTDT and CC (gTDT: RR = 0.54, CI = 0.29-1.00, *p* = 0.0511; mTDT: *p* = 0.0197; CC: OR = 0.61, CI = 0.39-0.92, *p* = 0.0162), suggesting that HLA-DRB1*01:01:01 may have protective effects also in the context of non HLA-DRB1*15:01 haplotypes. We did not find statistically significant protective effects mediated by the HLA-DRB1*01:01:01 allele in the context of the HLA-DPB1*104:01 risk effect described above.

The HLA-DRB3*02:02:01:02~HLA-DRB1*11:01:01:01~HLA-DQA1*05:05:01:01~HLA-DQB1*03:01:01:03 haplotype fragment showed moderate protective effects for gTDT, mTDT and CC (gTDT: RR = 0.51, CI = 0.32-0.81, *p* = 0.0043; mTDT: *p* = 0.0085; CC: OR = 0.66, CI = 0.44-0.97, *p* = 0.0325). Individual alleles and smaller haplotype segments showed similar protective effects (**Supplemental Table 6**).

Interestingly, we also observed statistically significant protective effects in the case-control analysis for the HLA-DRB4*01:03:01:01~HLA-DRB1*04:01:01:01~HLA-DQA1*03:03:01:01~HLA-DQB1*03:01:01:01 haplotype, which has been described before (16), but not in the gTDT and mTDT analyses (**Supplemental Table 6**). When these haplotypes were compared, we noticed the presence of a shared two-field HLA-DQB1*03:01 allele that showed protective effects for TDT and CC (**Table 2** and **Supplemental Table 6**). The results from the mTDT analysis were modestly statistically significant, suggesting altogether that the protective effects of HLA-DRB1*11:01:01:01 and HLA-DRB1*04:01:01:01 haplotypes are likely originated in the shared HLA-DQB1*03:01 allele.

A second HLA-DQB1*03 allele, HLA-DQB1*03:03 showed moderate protective effects in gTDT, mTDT and CC analyses (**Table 2** and **Supplemental Table 7**).

Finally, this dataset included 11 HLA-DPB1*09:01:01 parents from 11 trio families. Of these, only one parent transmitted HLA-DPB1*09:01:01 allele to an affected child. The allele HLA-DPB1*09:01:01 showed moderate protection assessed by gTDT, mTDT and CC tests (gTDT: RR = 0.1, CI = 0.01-0.78, *p* = 2.81e-02; mTDT: *p* = 3.22e-02; CC: OR = 0.15, CI = 0.00-0.92, *p* = 3.20e-02) (**Table 2** and **Supplemental Table 8**). Although the RR value for the allele HLA-DPB1*09:01:01 is low compared to the other protective alleles, the statistical significance is moderate due to the low frequency of the allele.

### Protective HLA Class I Alleles and Haplotype Blocks

The block including HLA-A*02:01:01:01~HLA-C*03:04:01:01~HLA-B*40:01:02 appears to be protective for MS (**Table 2** and **Supplemental Table 9**), this observation has been reported previously (5). We detected only 2 patients of the 252 that carried HLA-DRB5*01:01:01~HLA-DRB1*15:01:01:01 carrying also the protective HLA-A*02:01:01:01~HLA-C*03:04:01:01~HLA-B*40:01:02 block. When the allele names are truncated to two-field allele name, the statistical significance of the two-field HLA-A*02:01~HLA-C*03:04~HLA-B*40:01 haplotype was reduced (**Supplemental Table 9**) due to the presence of the less common haplotype (HLA-A*02:01:01:01~HLA-C*03:04:01:02~HLA-B*40:01:02). The difference between HLA-C*03:04:01:01 and HLA-C*03:04:01:02 is located in 3’-UTR (rs1049650).

The allele HLA-B*38:01 showed statistically significant protective effects (**Table 2** and **Supplemental Table 10**). This result is consistent with previous reports (4). The allele HLA-A*26:01 also showed similar statistical significance (**Supplemental Table 10**). In this study there were 60 trio families with at least one parent carrying the HLA-A*26:01 allele, and 44 families that had at least one parent carrying the HLA-B*38:01 allele. Of these families, 17 carried the HLA-A*26:01:01:01~HLA-C*12:03:01:01~HLA-B*38:01:01 haplotype. We performed HLA-A*26:01:01:01 and HLA-B*38:01:01 haplotype stratification analysis and the exclusion of families and individuals with these haplotypes/alleles, resulted in loss of the statistical significance associated with protection.

Another class I protective effect included the allele HLA-B*27:05:02 (**Table 2** and **Supplemental Table 11A**). Of 67 parental haplotypes with HLA-B*27:05:02, 21 haplotypes included the HLA-DRB1*01:01:01 allele, and 14 haplotypes included HLA-DQB1*03:01. We performed HLA-DRB1*01:01:01 haplotype stratification analysis which excluded trio families and individuals that contained the allele HLA-DRB1*01:01:01; the protective effect conferred by HLA-B*27:05:02 remained statistically significant. We performed a second haplotype stratification analysis by excluding trio families and individuals that carried the allele HLA-DQB1*03:01. The protective effects associated to HLA-B*27:05:02 remained statistically significant. Among 252 children who carried at least one haplotype bearing HLA-DRB1*15:01, we identified only 8 (3.2%) patients who had HLA-B*27:05:02 allele. Of 370 parents who had at least one haplotype bearing HLA-DRB1*15:01, we found 28 (7.6%) parents who had the allele HLA-B*27:05:02. These observations indicate the protective effects of HLA-B*27:05:02 in the presence of haplotypes bearing HLA-DRB1*15:01 (OR = 0.40; CI = 0.18-0.89; Yates *p* = 0.033306). When stratification analyses were performed by eliminating trio families with haplotypes bearing HLA-DRB1*15:01, no statistically significant under-transmission for HLA-B*27:05:02 was observed (**Supplemental Table 11B**). Conditional logistic regression TDT analysis confirmed a statistically significant interaction between the HLA-DRB5*01:01:01~HLA-DRB1*15:01:01:01 haplotype and the allele HLA-B*27:05:02 (RR = 0.23, CI = 0.1-0.51, 2 degree of freedom Wald test *p* = 1.29e-03).

A detailed transmission analysis was conducted for haplotype blocks in the extended family cases. Family 2965 exemplifies how protective and susceptibility alleles may interact (**Supplemental Figure 1**). This family had multiple generations of family members. All siblings in the first generation of the family carry haplotypes bearing the susceptibility associated block HLA-DRB5*01:01:01~HLA-DRB1*15:01:01:01; all the siblings not affected with MS carry a haplotype that includes HLA-B*27:02:01 while the affected subject inherited the alternative haplotype including HLA-B*08:01:01:01. This transmission pattern is consistent with HLA-B*27:02:01 disease-mediated protection in this particular family. The second-generation individual H0278DC4 diagnosed with MS inherited both, the protective HLA-B*27:02:01 allele and the high-risk HLA-DRB5*01:01:01~HLA-DRB1*15:01:01:01 haplotype block, further exemplifying the limited penetrance of protective and risk alleles and complex interaction underlying the association signals.

HLA-C*07:04 and HLA-B*44:02 showed statistically significant protective effects (**Table 2** and **Supplemental Table 12**). At four-field resolution, HLA-B*44:02 in European Americans presents two distinct common haplotypes: HLA-C*05:01:01:02~HLA-B*44:02:01:01 and HLA-C*07:04:01:01~HLA-B*44:02:01:03. The haplotype block of broadly defined alleles, HLA-C*05 and HLA-B*44, was reported as having a protective effect in MS in the absence of *HLA-DRB1* risk alleles (46–49). In the present study, no protective effect was observed for HLA-C*05:01. Although we observed higher significance of the protection by the allele HLA-C*07:04:01:01 (**Supplemental Table 12**), we could not rule out if this protection was associated with HLA-C*07:04 and/or HLA-B*44:02 due to the limited sample size and the tight LD between *HLA-C* and *HLA-B*.

### Assessing Risk and Protective Cumulative Effects

**Supplemental Table 13A** shows the risk and protective HLA allele/haplotype count summary in the case-control study data set. As expected, statistically significant more risk effects were observed in cases compared to no risk/protective factors present (**Supplemental Table 13B**: OR = 3.22, CI = 2.37-4.39, Yates *p* < 0.0001). The result confirms that subjects who carry the positive scores of the risk HLA alleles/haplotypes are more susceptible to MS than the subjects who do not carry risk and/or protective HLA alleles/haplotypes. Conversely, we observed statistically significant more protective effects in controls compared to no risk/protective factors present (**Supplemental Table 13C**: OR = 0.58, CI = 0.43-0.79, Yates *p* = 0.00060). In addition, aligned with the trio family results described above, the presence of risk factors predisposes to disease even in the presence of equal number of protective factors (**Supplemental Table 13D**: OR = 1.91, CI = 1.34-2.72, Yates *p* = 0.00042).

HLAGB scores (39, 40) were higher in cases (median [interquartile range (IQR)], 0.23 [-0.40 - 1.10]) compared to controls (median [IQR], -0.40 [−0.82 - 0.00]), fathers (median [IQR], -0.00 [−0.69 - 0.70]) and mothers (median [IQR], -0.00 [−0.80 - 0.56]) (**Supplemental Figure 2A**). We also observed statistically significant differences in HLAGB scores between the control group and parents (**Supplemental Figure 2A**). The control group shows lower HLAGB driven by higher burden of protective alleles compared to parents who carry the neutralized risk and protective factors. The over-transmission of risk alleles resulted in elevated HLA genetic burden predisposing to MS in their children. We did not find a statistically significant difference of HLAGB between parents (**Supplemental Figure 2A**), in gender of cases or controls (**Supplemental Figure 2BC**). Using the classification shown in **Supplemental Table 13A**, we observed statistically significant difference in both “Risk” and “Protective” groups of MS compared to controls (**Supplemental Figure 2D**).

## Discussion

In the present study, multiple HLA MS-risk and protective alleles were precisely mapped through the analysis of both, coding and non-coding variants. The HLA-DRB5*01:01:01~HLA-DRB1*15:01:01:01 block was observed in 52.8% of the affected children in the trio dataset, confirming the role of this block conferring susceptibility to MS at the four-field resolution (16). We were unable, however, to resolve the effect of individual alleles at *HLA-DRB1* and *HLA-DRB5* because of the exceptionally tight LD. The haplotype carrying HLA-DRB1*15:01 is the strongest genetic susceptibility factor for MS in Europeans (50), and has been reported as risk in Japanese (9) and African Americans (51). The etiologic role of HLA-DRB5*01:01 in MS was also demonstrated as a risk and disease modifier factor (52–54).

A novel independent association with susceptibility was identified for HLA-DPB1*104:01, observed in 6.1% of affected children. Interestingly, HLA-DPB1*03:01:01:01 and DPB1*104:01 share structural features, including identical exons 1 – 3 DNA sequences, but differ by a non-synonymous single nucleotide variation in exon 4 [rs11551421: GTG (Val) for HLA-DPB1*03:01 and ATG (Met) for HLA-DPB1*104:01], resulting in an amino acid difference located in the transmembrane domain GXXXG motif (GFVLG -> GFMLG) sequence (**Figure 1**). The allele frequencies of HLA-DPB1*03:01 and HLA-DPB1*104:01 were 0.08132 and 0.01824, respectively, in the control group. Noteworthy, these alleles cannot be distinguished using the conventional PCR-SSOP HLA typing method, and are currently reported in reference registries as HLA-DPB1*03:CUWXP, providing an explanation for this allele going undiscovered in that previous HLA-based analysis or studies with limited genotyping resolution.

Although this variation was reported to have a limited functional role in allorecognition of HLA-DPB1*03:01/HLA-DPB1*104:01 in unrelated stem cell donor selection (55), little is known about changes in the downstream immunological response (56, 57). HLA-DPB1*104:01 and HLA-DPB1*03:01:01 present additional differences in the non-coding regions and form two distinct haplotypes with specific *HLA-DPA1* alleles that differ in the fourth-field of otherwise, identical amino acid sequences (HLA-DPA1*01:03:01:02~HLA-DPB1*104:01 and HLA-DPA1*01:03:01:03~HLA-DPB1*03:01:01). We compared the HLA-DPA1*01:03:01:02 and HLA-DPB1*104:01 reference sequences from IPD-IMGT/HLA database with publicly available reference HLA-DPA1*01:03:01:03~HLA-DPB1*03:01:01 genomic sequences (42) and identified 26 single nucleotide polymorphisms (SNPs) between these haplotypes within the imputed DNA sequence region. Of these, 22 SNPs are located in intron 1 of *HLA-DPB1* (**Figure 1**). The *HLA-DPA1* promoter is located in the complementary strand of genomic DNA sequence that overlaps with intron 1 and exon 2 of *HLA-DPB1* (**Figure 1**) (58). Polymorphisms in promoters in *HLA-DPB1* have been associated with autoimmune disease such as systemic sclerosis (59). We identified four SNPs (rs3135020, rs3097670, rs3130169 and rs3128959) that are associated with expression of brain cerebellum for *HLA-DPA1*, and brain nucleus accumbens (basal ganglia) for *HLA-DPB1*. The later three SNPs are located within the *HLA-DPA1* promoter region or *HLA-DPB1* intron 1 (**Figure 1**) (58). Seven different HLA-DPB1*104:01 alleles that includes intron 1 sequences are recognized as of March 2021, and all alleles share the same nucleotide at three eQTL associated SNPs (rs3097670, rs3130169 and rs3128959) (**Supplemental Figure 3**). If these eQTLs combined with the structural features for antigen presentation by HLA-DPB1*104:01 were to confer susceptibility, it can be speculated that any of these variants could be involved in MS pathogenesis.

In the class II region, we found one HLA-DRB1 and two HLA-DQB1 protein variants conferring protection to developing MS. The allele HLA-DRB1*01:01:01 appeared to be the principal protective determinant in haplotypes bearing this allele. The examination of interactions between protective and susceptibility alleles, indicate that HLA-DRB1*01:01:01 confers dominant protection. Our analysis is also consistent with protective effects by HLA-DQB1*03:01 and HLA-DQB1*03:03. Furthermore, the HLA-DRB1*09:01 allele reported to be protective in Asian populations (8, 9) may have derived from the primary association with HLA-DQB1*03:03 (60, 61). We performed pairwise comparisons of the amino acid sequences of the alleles HLA-DQB1*03:01, HLA-DQB1*03:02 and HLA-DQB1*03:03. The allele HLA-DQB1*03:01 differs from HLA-DQB1*03:02 and HLA-DQB1*03:03 by replacements in 7 and 6 amino acids with 4 and 3 substitutions at residues located at the peptide binding domain (PDB), respectively. In contrast, HLA-DQB1*03:02 and HLA-DQB1*03:03 differ only by the replacement of one amino acid at residue 57 located at the PDB. The alleles associated with protection present the same charged amino acid (Aspartic acid) at this residue while the non-protective allele carries the hydrophobic Alanine. The amino acid substitutions at residue 57 of DQB1 have been postulated to play important roles in susceptibility and resistance in insulin-dependent diabetes mellitus (IDDM) (62).

The allele HLA-B*44:02 is frequently associated with the protective allele HLA-DQB1*03:01, thus protection associated with these alleles was difficult to resolve. The neutral transmission of haplotypes bearing both HLA-B*44:02 and the highly susceptibility allele HLA-DRB1*15:01:01:01 is suggestive that the observed susceptibility conferred by HLA-DRB1*15:01:01:01 over-transmitted in haplotypes bearing multiple alleles of *HLA-B* is specifically neutralized by the presence of HLA-B*44:02. We conclude that the HLA-B*44:02 protein effect in protection is likely to be an independent factor. Conditional logistic regression TDT analysis suggested a statistically significant interaction between the haplotype carrying HLA-DRB1*15:01 and HLA-B*44:02 (RR = 0.55, CI = 0.32-0.94, 2 degree of freedom Wald test *p* = 3.05e-02).

The HLA allele transmission analysis in family 2965 provides an interesting insight of a protective factor offsetting the effect of a susceptibility allele (**Supplemental Figure 1**). The observation of protection conferred by HLA-B*27:05:02 and HLA-B*27:02:01 suggests that antigen presenting features of this allele and closely related alleles may confer protection. The HLA-B*27:05 and HLA-B*27:02 are both serologically recognized as B27, and carry the Bw4 ligand for a killer immunoglobulin-like receptor KIR3DL1. The KIR3DL1 in combination with Bw4 epitope was shown to be protective against MS in African Americans (63). We performed gTDT with the first-field allele name (serological equivalent) for the 477 trios and observed the statistical significance of HLA-B*27 (gTDT: RR = 0.37, CI = 0.21-0.63, *p* = 0.00028) (17).

In the case of protection conferred by the class I haplotype HLA-A*02:01:01:01~HLA-B*40:01:02~HLA-C*03:04:01:01, we hypothesize that these three HLA alleles are not the protective factors *per se*, and other elements exert protection within the 1.45 Mb genomic region that is specific to this class I haplotype block.

In summary, HLA-DRB5*01:01:01~ HLA-DRB1*15:01:01:01 was significantly associated with predisposition as expected. A second independent risk allele, HLA-DPB1*104:01 was newly identified. HLA-DRB1*01:01:01, HLA-DQB1*03:01 and HLA-DQB1*03:03, showed protective effects. The HLA class I block, HLA-A*02:01:01:01~ HLA-C*03:04:01:01~ HLA-B*40:01:02 and the alleles HLA-B*44:02, HLA-B*27:05 and HLA-B*38:01 showed moderately protective effects independently from each other and from the class II associated factors. Altogether, the present study demonstrates the effectiveness of high resolution extended coverage typing for dissecting HLA alleles/haplotypes associated with disease susceptibility using family-based segregation analyses. This is the first MS-HLA family study using NGS. In this study both, susceptible and protective candidate HLA alleles/haplotypes were mapped with more precision by eliminating at the same time false signals resulting from ‘hitchhiking’ alleles (64). The mapping to specific HLA allele structures may allow to design research focused in their functional features facilitating the understanding of the mechanisms involved in disease susceptibility and protection. In addition, the HLA genetic burden (HLAGB) defined by HLA genotype evaluated in this study appears to generate a highly informative risk score (RS) that could be further evaluated in the outcomes of the disease in future studies.

## Data Availability Statement

The original contributions presented in the study are included in the article/**Supplementary Material**. Further inquiries can be directed to the corresponding author.

## Ethics Statement

The studies involving human participants were reviewed and approved by The University of California, San Francisco Institutional Review Board & the Stanford University Institutional Review Board. The patients/participants provided their written informed consent to participate in this study.

## Author Contributions

This study was conceived and designed by KO, MF, and JO. KO conducted data quality control and performed the statistical analysis. KO, MF, and JO were also involved in data interpretation and drafting the manuscript. LC, GM-M, KM, and SG were responsible for the NGS genotyping assays. NI developed the HLGB scores. JH contributed to the analysis plan. SH was responsible for the collection of samples and clinical data. SC and AS were responsible for sample and data management at the UCSF-DNA bank. All authors contributed to the article and approved the submitted version.

## Funding

This study was supported by a grant from the National Institutes of Health U19NS095774 (JO and MV). The UCSF DNA biorepository is supported by RG-1611-26299 from the National Multiple Sclerosis Society. The content is solely the responsibility of the authors and does not necessarily reflect the official views of the NIAID, NINDS, NIH or United States Government.

## Conflict of Interest

The authors declare that the research was conducted in the absence of any commercial or financial relationships that could be construed as a potential conflict of interest.
