# Supplementary material for: High Resolution Haplotype Analyses of Classical HLA Genes in Families With Multiple Sclerosis Highlights the Role of HLA-DP Alleles in Disease Susceptibility
Source: Front Immunol. 2021 May 25;12:644838. doi: 10.3389/fimmu.2021.644838 (PMC8240666; doi:10.3389/fimmu.2021.644838)
Supplement: Supplementary file 1 [file DataSheet_1.docx]

Supplemental Figure 1: Family 2965


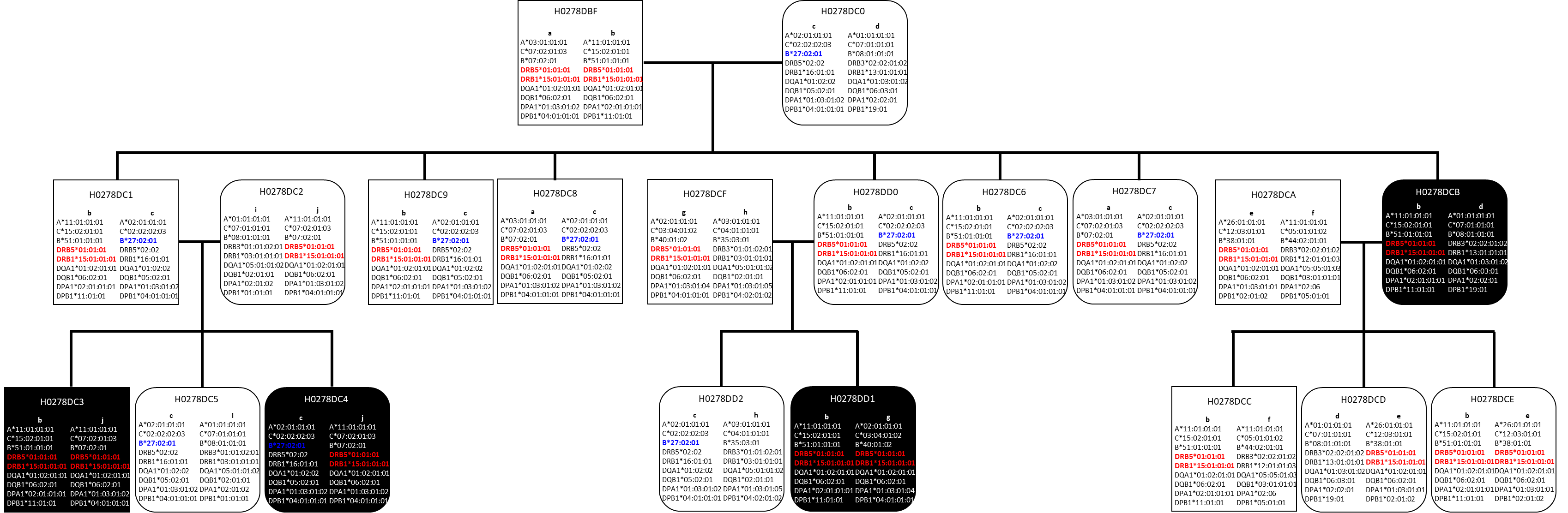


The square shape shows male, and rounded-corner square indicate female. Individuals who are diagnosed with MS are shown in black-filled shape, while non-filled shapes indicates unaffected. The prefix “HLA-“ is omitted from the HLA allele names. No DNA specimen was available for H0278DBF, H0278DC0, H0278DCA and H0278DCB. The HLA genotypes of these subjects were assigned unequivocally, because specimens from their children were tested and allowed for the analyses of transmitted haplotypes. An individual (H0278DBF) who has no MS carries homozygous risk haplotype bearing *HLA-DRB1*15:01*. There were 7 siblings in the first generation of the family, and only one of them was affected. All 7 children inherited the identical risk haplotype from H0278DBF. Six children who were not diagnosed with MS inherited the same haplotype from H0278DC0. Only one child (H0278DCB) diagnosed with MS received the second haplotype from H0278DC0. We speculate that H0278DCB developed MS, because the subject did not receive a protective haplotype while the other six non-MS children received protective haplotype. The risk haplotypes are indicated in red, and putative protective allele is shown in blue.

Supplemental Figure 2

A: HLAGB Distribution


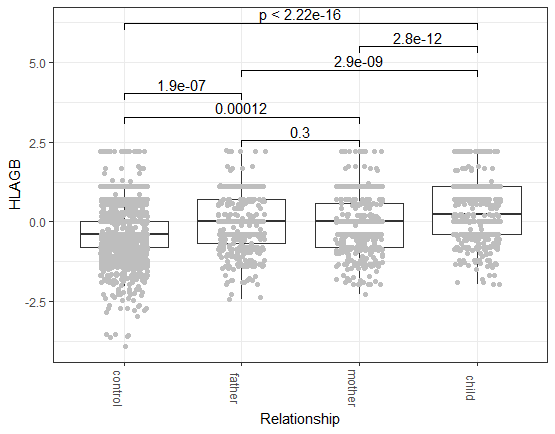


B: MS cases


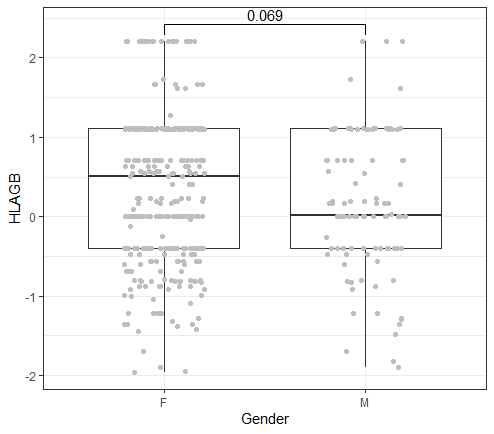


C: Controls


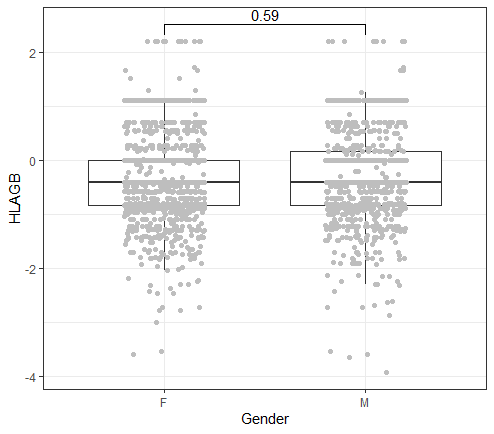


D: MS vs. Controls


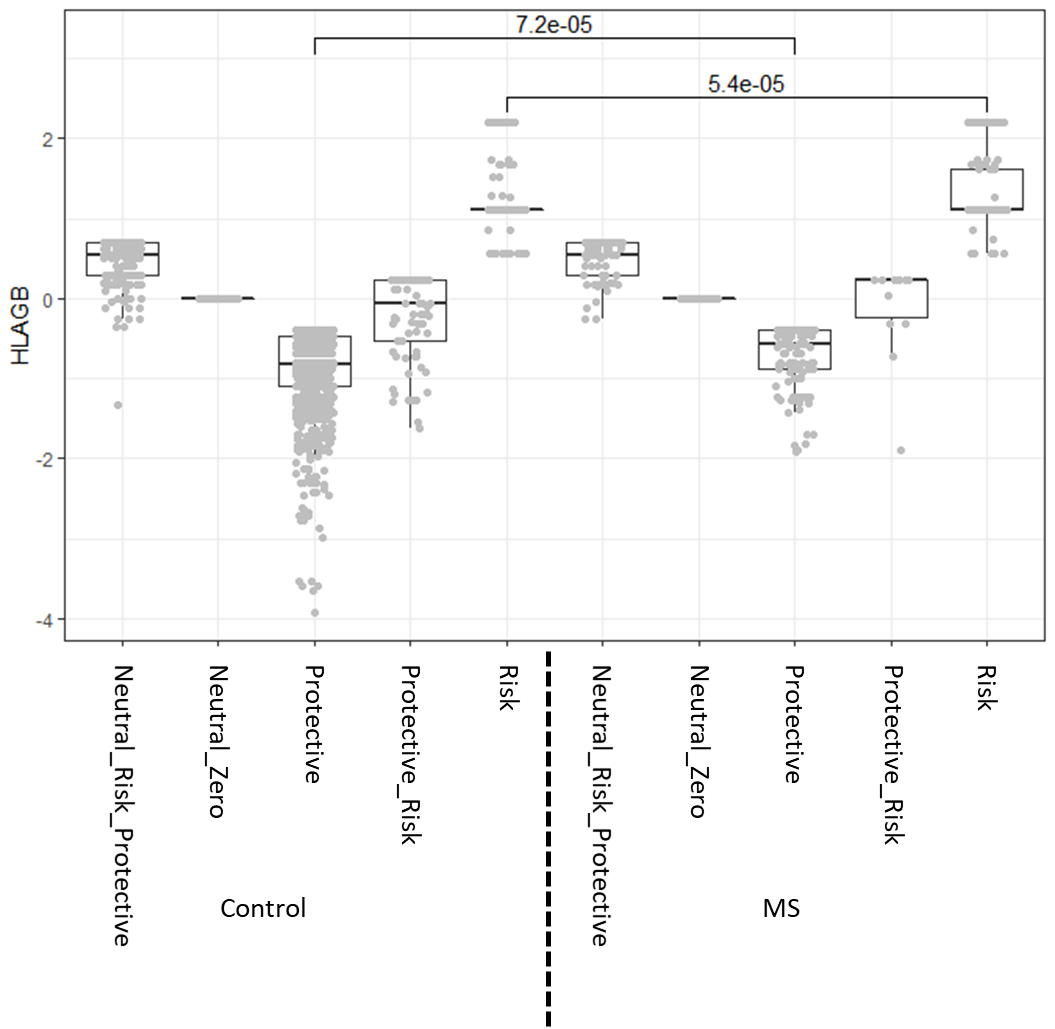


Supplemental Figure 2: HLAGB score distribution.

The Supplemental Figure 2A shows HLAGB score distribution for cases (children), father, mother and controls using box plots. Bold horizontal lines in the boxes represent median values and the heights of the boxes show the interquartile range (IQR). A Wilcoxon test p-values are shown for the comparison. Statistically significant differences are observed between cases and parents or cases and controls.

The HLAGB scores were plotted for male and female of patients with multiple sclerosis (MS) (2B) and controls (2C) using box plots. Bold horizontal lines in the boxes represent median values and the heights of the boxes show the interquartile range (IQR). A Wilcoxon test *p*-value is shown for the comparison. No significant difference was observed for HLAGB scores by sexes in both case (2B) and control (2C) groups. Figure 2D shows HLAGB score distribution for five groups of MS and Control: Risk group, Neutral_Risk_Protective (equivalent numbers of risk and protective factors), Neutral_Zero risk (no risk and protective factors present), Protective_Risk (negative score, but contains at least one risk allele) and Protective (negative score).

Supplemental Figure 3: DNA sequence alignment of *HLA-DPB1*03:01:01:01* with *HLA-DPB1*104:01* alleles.

gDNA 2910 2920 2930 2940 2950 2960 2970 2980 2990 3000

DPB1*03:01:01:01 GGGGGCCCTG GGCCTGGAAT TTCAGGGTCT GGCGCCCAAG GCACCAGGAG AAGAGGCAGG TCAGGATATC TGAGTCAAGA CCTGGGATCT TGCCTTAGCA

DPB1*104:01:01:01 ---------- ---------- ---------- --G------- ---------- ---------- ---------- ---------- ---------- ----------

DPB1*104:01:01:02 ---------- ---------- ---------- --G------- ---------- ---------- ---------- ---------- ---------- ----------

DPB1*104:01:01:03 ---------- ---------- ---------- --G------- ---------- ---------- ---------- ---------- ---------- ----------

DPB1*104:01:01:04 ---------- ---------- ---------- --G------- ---------- ---------- ---------- ---------- ---------- ----------

DPB1*104:01:01:05 ---------- ---------- ---------- --G------- ---------- ---------- ---------- ---------- ---------- ----------

DPB1*104:01:01:06 ---------- ---------- ---------- --G------- ---------- ---------- ---------- ---------- ---------- ----------

DPB1*104:01:02 ---------- ---------- ---------- --G------- ---------- ---------- ---------- ---------- ---------- ----------

rs3097670

gDNA 3010 3020 3030 3040 3050 3060 3070 3080 3090 3100

DPB1*03:01:01:01 ATGACACTGG AGACTAAAGG TGGACTCCAT GGTGCCCTTG AGCCCAGCCC TACCCCATCT CCACTATCCT CTGCCACCAG CTGTGCAACT TCTGCAAGGG

DPB1*104:01:01:01 ---------- ---------- ---------- ---------- ---------- ---------- ---------- ---------- ---------- ----------

DPB1*104:01:01:02 ---------- ---------- ---------- ---------- ---------- ---------- ---------- ---------- ---------- ----------

DPB1*104:01:01:03 ---------- ---------- ---------- ---------- ---------- ---------- ---------- ---------- ---------- ----------

DPB1*104:01:01:04 ---------- ---------- ---------- ---------- ---------- ---------- ---------- ---------- ---------- ----------

DPB1*104:01:01:05 ---------- ---------- ---------- ---------- ---------- ---------- ---------- ---------- ---------- ----------

DPB1*104:01:01:06 ---------- ---------- ---------- ---------- ---------- ---------- ---------- ---------- ---------- ----------

DPB1*104:01:02 ---------- ---------- ---------- ---------- ---------- ---------- ---------- ---------- ---------- ----------

gDNA 3110 3120 3130 3140 3150 3160 3170 3180 3190 3200

DPB1*03:01:01:01 GTGAGGTTAA TAAACTGGAG AAGTTAATTT GTGGAGCATG AAACAGATGA GCAGAACAAT CACAGCACCT TAATTTCCCC AGTGTGCCCA AGAACAGAGC

DPB1*104:01:01:01 ---------- ---------- ---------- ---------- ---------- ---------- ---------- ---------- ---------- ----------

DPB1*104:01:01:02 ---------- ---------- ---------- ---------- ---------- ---------- ---------- ---------- ---------- ----------

DPB1*104:01:01:03 ---------- ---------- ---------- ---------- ---------- ---------- ---------- ---------- ---------- ----------

DPB1*104:01:01:04 ---------- ---------- ---------- ---------- ---------- ---------- ---------- ---------- ---------- ----------

DPB1*104:01:01:05 ---------- ---------- ---------- ---------- ---------- ---------- ---------- ---------- ---------- ----------

DPB1*104:01:01:06 ---------- ---------- ---------- ---------- ---------- ---------- ---------- ---------- ---------- ----------

DPB1*104:01:02 ---------- ---------- ---------- ---------- ---------- ---------- ---------- ---------- ---------- ----------

gDNA 3210 3220 3230 3240 3250 3260 3270 3280 3290 3300

DPB1*03:01:01:01 AGGCCTGAAG ACACTCAAAC AGAAACAAAC ATGTGCCGTG TCACTGATAA TTCTGTGTAG ACACACACCT GCCAGACACT GCTCATGGCA CTCCCTAGGA

DPB1*104:01:01:01 ---------- ---------- ---------- ---------- ---------- ---------- ---------- ---------- ---------- ----------

DPB1*104:01:01:02 ---------- ---------- ---------- ---------- ---------- ---------- ---------- ---------- ---------- ----------

DPB1*104:01:01:03 ---------- ---------- ---------- ---------- ---------- ---------- ---------- ---------- ---------- ----------

DPB1*104:01:01:04 ---------- ---------- ---------- ---------- ---------- ---------- ---------- ---------- ---------- ----------

DPB1*104:01:01:05 ---------- ---------- ---------- ---------- ---------- ---------- ---------- ---------- ---------- ----------

DPB1*104:01:01:06 ---------- ---------- ---------- ---------- ---------- ---------- ---------- ---------- ---------- ----------

DPB1*104:01:02 ---------- ---------- ---------- ---------- ---------- ---------- ---------- ---------- ---------- ----------

gDNA 3310 3320 3330 3340 3350 3360 3370 3380 3390 3400

DPB1*03:01:01:01 AGAACAGCAT GTGGGAAAGG CTGCCAAAAT TGTTCATGTA AAAATTACAT CAATGCTGTC TTCCTCGGTG CTGCCTATGC AGCTGGCAGC CATCTCTTCC

DPB1*104:01:01:01 ---------- ---------- ---------- ---------- ---------- ---------- ---------- ---------- ---------- ----------

DPB1*104:01:01:02 ---------- ---------- ---------- ---------- ---------- ---------- ---------- ---------- ---------- ----------

DPB1*104:01:01:03 ---------- ---------- ---------- ---------- ---------- ---------- ---------- ---------- ---------- ----------

DPB1*104:01:01:04 ---------- ---------- ---------- ---------- ---------- ---------- ---------- ---------- ---------- ----------

DPB1*104:01:01:05 ---------- ---------- ---------- ---------- ---------- ---------- ---------- ---------- ---------- ----------

DPB1*104:01:01:06 ---------- ---------- ---------- ---------- ---------- ---------- ---------- ---------- ---------- ----------

DPB1*104:01:02 ---------- ---------- ---------- ---------- ---------- ---------- ---------- ---------- ---------- ----------

gDNA 3410 3420 3430 3440 3450 3460 3470 3480 3490 3500

DPB1*03:01:01:01 TCCACATCAT GGCCTCCCTC AGACTCCTCA TGAAGGATAA GATCCTCAAA AAGAGGACCA ACAAGTTCAT GAGGCACCAA TCAGACTGAA ATGTCAAAAT

DPB1*104:01:01:01 ---------- ---------- ---------- ---------- ---------- ---------- ---------- ---------- ---------- ----------

DPB1*104:01:01:02 ---------- ---------- ---------- ---------- ---------- ---------- ---------- ---------- ---------- ----------

DPB1*104:01:01:03 ---------- ---------- ---------- ---------- ---------- ---------- ---------- ---------- ---------- ----------

DPB1*104:01:01:04 ---------- ---------- ---------- ---------- ---------- ---------- ---------- ---------- ---------- ----------

DPB1*104:01:01:05 ---------- ---------- ---------- ---------- ---------- ---------- ---------- ---------- ---------- ----------

DPB1*104:01:01:06 ---------- ---------- ---------- ---------- ---------- ---------- ---------- ---------- ---------- ----------

DPB1*104:01:02 ---------- ---------- ---------- ---------- ---------- ---------- ---------- ---------- ---------- ----------

gDNA 3510 3520 3530 3540 3550 3560 3570 3580 3590 3600

DPB1*03:01:01:01 TAAGCATAAC TGGCGGAAAC CCAGAGGTCT TAACAGTAGG GTTCGTAGAA GGTCCAAGGG CCAGATCTTG ATGCCCAACA TTGCTTATGG GAGCAACAAC

DPB1*104:01:01:01 ---------- ---------- ---------- ---------- ---------- ---------- ---------- ---------- ---------- ----------

DPB1*104:01:01:02 ---------- ---------- ---------- ---------- ---------- ---------- ---------- ---------- ---------- ----------

DPB1*104:01:01:03 ---------- ---------- ---------- ---------- ---------- ---------- ---------- ---------- ---------- ----------

DPB1*104:01:01:04 ---------- ---------- ---------- ---------- ---------- ---------- ---------- ---------- ---------- ----------

DPB1*104:01:01:05 ---------- ---------- ---------- ---------- ---------- ---------- ---------- ---------- ---------- ----------

DPB1*104:01:01:06 ---------- ---------- ---------- ---------- ---------- ---------- ---------- ---------- ---------- ----------

DPB1*104:01:02 ---------- ---------- ---------- ---------- ---------- ---------- ---------- ---------- ---------- ----------

gDNA 3610 3620 3630 3640 3650 3660 3670 3680 3690 3700

DPB1*03:01:01:01 AACAACAAAA AACACATGCT GCCCAGTGGC TTCCAGAAGT TTCTGGCCCA CAGCCTCAAG GAGCTGAAAG TGCTGCTGAT GTGCAACAAA TCTTACTGTG

DPB1*104:01:01:01 ---------- ---------- ---------- ---------- ---------- ---------- ---------- ---------- ---------- ----------

DPB1*104:01:01:02 ---------- ---------- ---------- ---------- ---------- ---------- ---------- ---------- ---------- ----------

DPB1*104:01:01:03 ---------- ---------- ---------- ---------- ---------- ---------- ---------- ---------- ---------- ----------

DPB1*104:01:01:04 ---------- ---------- ---------- ---------- ---------- ---------- ---------- ---------- ---------- ----------

DPB1*104:01:01:05 ---------- ---------- ---------- ---------- ---------- ---------- ---------- ---------- ---------- ----------

DPB1*104:01:01:06 ---------- ---------- ---------- ---------- ---------- ---------- ---------- ---------- ---------- ----------

DPB1*104:01:02 ---------- ---------- ---------- ---------- ---------- ---------- ---------- ---------- ---------- ----------

gDNA 3710 3720 3730 3740 3750 3760 3770 3780 3790 3800

DPB1*03:01:01:01 CTGAGATCGC TCACAAAATT TCCTCCAGAA CTGCAAAGTC ATCATGGAAA GAGTCACCCA GCCGGCCATC AGAGTCACCA ACCCCAGTAC CACGGTGCAC

DPB1*104:01:01:01 ---------- ---------- ---------- ---------- ---------- ---------- ---------- ---------- ---------- ----------

DPB1*104:01:01:02 ---------- ---------- ---------- ---------- ---------- ---------- ---------- ---------- ---------- ----------

DPB1*104:01:01:03 ---------- ---------- ---------- ---------- ---------- ---------- ---------- ---------- ---------- ----------

DPB1*104:01:01:04 ---------- ---------- ---------- ---------- ---------- ---------- ---------- ---------- ---------- ----------

DPB1*104:01:01:05 ---------- ---------- ---------- ---------- ---------- ---------- ---------- ---------- ---------- ----------

DPB1*104:01:01:06 ---------- ---------- ---------- ---------- ---------- ---------- ---------- ---------- ---------- ----------

DPB1*104:01:02 ---------- ---------- ---------- ---------- ---------- ---------- ---------- ---------- ---------- ----------

gDNA 3810 3820 3830 3840 3850 3860 3870 3880 3890 3900

DPB1*03:01:01:01 AGCTAAGAAA ATGAGTAGAA AGTTCATGTC CACGTTTTGT GTGTAAATAA AACCATAAAA ACTGCCAAAA AAAATTACAT CAATGCCTCT AAACCCAAAG

DPB1*104:01:01:01 ---------- ---G------ ---------- ---------- ---------- ---------- ---------- ---------- ---------- ----------

DPB1*104:01:01:02 ---------- ---G------ ---------- ---------- ---------- ---------- ---------- ---------- ---------- ----------

DPB1*104:01:01:03 ---------- ---G------ ---------- ---------- ---------- ---------- ---------- ---------- ---------- ----------

DPB1*104:01:01:04 ---------- ---G------ ---------- ---------- ---------- ---------- ---------- ---------- ---------- ----------

DPB1*104:01:01:05 ---------- ---G------ ---------- ---------- ---------- ---------- ---------- ---------- ---------- ----------

DPB1*104:01:01:06 ---------- ---G------ ---------- ---------- ---------- ---------- ---------- ---------- ---------- ----------

DPB1*104:01:02 ---------- ---G------ ---------- ---------- ---------- ---------- ---------- ---------- ---------- ----------

gDNA 3910 3920 3930 3940 3950 3960 3970 3980 3990 4000

DPB1*03:01:01:01 GACTCTACCC CCACAGGTCC CTGGTTGTTG TGGTGATTTT CATTGTGTAA AATACTTTCC ACATCTTTTG ACACCAAGTC TTTCTGCAGC CATGTTTGAA

DPB1*104:01:01:01 ---------- ---------- ---------- ---------- ---------- ---------- ---------- ---------- ---------- ----------

DPB1*104:01:01:02 ---------- ---------- ---------- ---------- ---------- ---------- ---------- ---------- ---------- ----------

DPB1*104:01:01:03 ---------- ---------- ---------- ---------- ---------- ---------- ---------- ---------- ---------- ----------

DPB1*104:01:01:04 ---------- ---------- ---------- ---------- ---------- ---------- ---------- ---------- ---------- ----------

DPB1*104:01:01:05 ---------- ---------- ---------- ---------- ---------- ---------- ---------- ---------- ---------- ----------

DPB1*104:01:01:06 ---------- ---------- ---------- ---------- ---------- ---------- ---------- ---------- ---------- ----------

DPB1*104:01:02 ---------- ---------- ---------- ---------- ---------- ---------- ---------- ---------- ---------- ----------

gDNA 4010 4020 4030 4040 4050 4060 4070 4080 4090 4100

DPB1*03:01:01:01 AATTAACTTT CAGGCTACAG AGTCTTTCTT ATACCAAAGT TGAAGAAAGT TTTAAGAAAT ATATTTCTAC ATCTCCTACA TGCAAAACAA CAGGAGCAAG

DPB1*104:01:01:01 ---------- ---------- ---------- ---------- ---------- ---------- ---------- ---------- ---------- ----------

DPB1*104:01:01:02 ---------- ---------- ---------- ---------- ---------- ---------- ---------- ---------- ---------- ----------

DPB1*104:01:01:03 ---------- ---------- ---------- ---------- ---------- ---------- ---------- ---------- ---------- ----------

DPB1*104:01:01:04 ---------- ---------- ---------- ---------- ---------- ---------- ---------- ---------- ---------- ----------

DPB1*104:01:01:05 ---------- ---------- ---------- ---------- ---------- ---------- ---------- ---------- ---------- ----------

DPB1*104:01:01:06 ---------- ---------- ---------- ---------- ---------- ---------- ---------- ---------- ---------- ----------

DPB1*104:01:02 ---------- ---------- ---------- ---------- ---------- ---------- ---------- ---------- ---------- ----------

gDNA 4110 4120 4130 4140 4150 4160 4170 4180 4190 4200

DPB1*03:01:01:01 TTGAGGAATT CTCAAGAAAC TGGTCGAGAA GAGAGAGCGC TTAGCTATGG AAAAGAGAAA GAAGGAAGGG AGGGCTTCCT GGAGGAGGTG GCATTTGAAC

DPB1*104:01:01:01 ---------- ---------- ---------- ---------- ---------- ---------- ---------- ---------- ---------- ----------

DPB1*104:01:01:02 ---------- ---------- ---------- ---------- ---------- ---------- ---------- ---------- ---------- ----------

DPB1*104:01:01:03 ---------- ---------- ---------- ---------- ---------- ---------- ---------- ---------- ---------- ----------

DPB1*104:01:01:04 ---------- ---------- ---------- ---------- ---------- ---------- ---------- ---------- ---------- ----------

DPB1*104:01:01:05 ---------- ---------- ---------- ---------- ---------- ---------- ---------- ---------- ---------- ----------

DPB1*104:01:01:06 ---------- ---------- ---------- ---------- ---------- ---------- ---------- ---------- ---------- ----------

DPB1*104:01:02 ---------- ---------- ---------- ---------- ---------- ---------- ---------- ---------- ---------- ----------

gDNA 4210 4220 4230 4240 4250 4260 4270 4280 4290 4300

DPB1*03:01:01:01 CAGGACTGAC ATCAGGATGG AAATGTCAGT CAGGGAGTTA AGTAGGGGGA GCAGCTCCGC CCTCCACGTC CCCAGCTCCT CCTGCCCCTG TTTTTTCTCC

DPB1*104:01:01:01 ---------- ---------- ---------- ---------- ---------- ---------- ---------- ---------- --C------- ----------

DPB1*104:01:01:02 ---------- ---------- ---------- ---------- ---------- ---------- ---------- ---------- --C------- ----------

DPB1*104:01:01:03 ---------- ---------- ---------- ---------- ---------- ---------- ---------- ---------- --C------- ----------

DPB1*104:01:01:04 ---------- ---------- ---------- ---------- ---------- ---------- ---------- ---------- --C------- ----------

DPB1*104:01:01:05 ---------- ---------- ---------- ---------- ---------- ---------- ---------- ---------- --C------- ----------

DPB1*104:01:01:06 ---------- ---------- ---------- ---------- ---------- ---------- ---------- ---------- --C------- ----------

DPB1*104:01:02 ---------- ---------- ---------- ---------- ---------- ---------- ---------- ---------- --C------- ----------

rs3130169

gDNA 4310 4320 4330 4340 4350 4360 4370 4380 4390 4400

DPB1*03:01:01:01 CAGTGACCCC ACGTGAAACG TCTCCGCCTC CTCCAGCCAC CAGCAGAAGG GACTGCCTTC CCCTCAGTGC TCGCCCCTCC CTAGTGATCA CTCAGTGCCC

DPB1*104:01:01:01 ---------- ---------- ---------- ---------- ---------- ---------- ---------- ---------- ---------- ----------

DPB1*104:01:01:02 ---------- ---------- ---------- ---------- ---------- ---------- ---------- ---------- ---------- ----------

DPB1*104:01:01:03 ---------- ---------- ---------- ---------- ---------- ---------- ---------- ---------- ---------- ----------

DPB1*104:01:01:04 ---------- ---------- ---------- ---------- ---------- ---------- ---------- ---------- ---------- ----------

DPB1*104:01:01:05 ---------- ---------- ---------- ---------- ---------- ---------- ---------- ---------- ---------- ----------

DPB1*104:01:01:06 ---------- ---------- ---------- ---------- ---------- ---------- ---------- ---------- ---------- ----------

DPB1*104:01:02 ---------- ---------- ---------- ---------- ---------- ---------- ---------- ---------- ---------- ----------

gDNA 4410 4420 4430 4440 4450 4460 4470 4480 4490 4500

DPB1*03:01:01:01 CTGAGCTCAT TCTTTTCAGT AAATTCTCTC TCTGCGTGGT GAGAAAACAG GCCTGGAGAG GCTCTGCGAC CCGCTTAGGA CCACAGAACT CGGTACTAGG

DPB1*104:01:01:01 ---------- ---------- ---------- ---------- ---------- ---------- ---------- ---------- ---------- ----------

DPB1*104:01:01:02 ---------- ---------- ---------- ---------- ---------- ---------- ---------- ---------- ---------- ----------

DPB1*104:01:01:03 ---------- ---------- ---------- ---------- ---------- ---------- ---------- ---------- ---------- ----------

DPB1*104:01:01:04 ---------- ---------- ---------- ---------- ---------- ---------- ---------- ---------- ---------- ----------

DPB1*104:01:01:05 ---------- ---------- ---------- ---------- ---------- ---------- ---------- ---------- ---------- ----------

DPB1*104:01:01:06 ---------- ---------- ---------- ---------- ---------- ---------- ---------- ---------- ---------- ----------

DPB1*104:01:02 ---------- ---------- ---------- ---------- ---------- ---------- ---------- ---------- ---------- ----------

gDNA 4510 4520 4530 4540 4550 4560 4570 4580 4590 4600

DPB1*03:01:01:01 AAAACTCCTA TTTTAAAATC CAGCCCTGGG TGGGAAGATT TGGGAAGAAT CGTTAATATT AAGAGAGAGA GGGAGAAAGA GGATTAGATG AGAGTGGCGC

DPB1*104:01:01:01 ---------- ---------- ---------- ---------- ---------- ---------- G--------- ---------- ---------- ----------

DPB1*104:01:01:02 ---------- ---------- ---------- ---------- ---------- ---------- G--------- ---------- ---------- ----------

DPB1*104:01:01:03 ---------- ---------- ---------- ---------- ---------- ---------- G--------- ---------- ---------- ----------

DPB1*104:01:01:04 ---------- ---------- ---------- ---------- ---------- ---------- G--------- ---------- ---------- ----------

DPB1*104:01:01:05 ---------- ---------- ---------- ---------- ---------- ---------- G--------- ---------- ---------- ----------

DPB1*104:01:01:06 ---------- ---------- ---------- ---------- ---------- ---------- G--------- ---------- ---------- ----------

DPB1*104:01:02 ---------- ---------- ---------- ---------- ---------- ---------- G--------- ---------- ---------- ----------

rs3128959

gDNA 4610 4620

DPB1*03:01:01:01 CTCCGCTCAT GTCCGCCCCC TCCCCGCAG

DPB1*104:01:01:01 ---------- ---------- ---------

DPB1*104:01:01:02 ---------- ---------- ---------

DPB1*104:01:01:03 ---------- ---------- ---------

DPB1*104:01:01:04 ---------- ---------- ---------

DPB1*104:01:01:05 ---------- ---------- ---------

DPB1*104:01:01:06 ---------- ---------- ---------

DPB1*104:01:02 ---------- ---------- ---------

DNA sequence alignment of HLA-DPB1*03:01:01:01 and HLA-DPB1*104:01 alleles. Partial DNA sequence of HLA-DPB1*03:01:01:01 is shown in this figure. The three eQTL associated SNP (rs3097670, rs3130169 and rs3128959) are shown in red.
