# Supplementary material for: High Resolution Haplotype Analyses of Classical HLA Genes in Families With Multiple Sclerosis Highlights the Role of HLA-DP Alleles in Disease Susceptibility
Source: Front Immunol. 2021 May 25;12:644838. doi: 10.3389/fimmu.2021.644838 (PMC8240666; doi:10.3389/fimmu.2021.644838)
Supplement: Supplementary file 2 [file Table_1.docx]

Supplemental Table 1: Clinical and demographic information in MS patient from extended families

|  | Quartet  Father + Daughter | Quartet  Father + Son | Quartet  Mother + Daughter | Quartet  Mother + Son | Cousins | Multi-generation - other | Siblings + | Total |
| --- | --- | --- | --- | --- | --- | --- | --- | --- |
| Families | 10 | 2 | 20 | 6 | 5 | 3 | 8 | 54 |
| N | 50 | 9 | 121 | 33 | 63 | 29 | 105 | 410 |
| Mean samples / family | 5 | 4.5 | 6.1 | 5.5 | 12.6 | 9.7 | 13.1 | 7.6 |
| MS (F:M) | 22 (1:1) | 5 (0:1) | 55 (8.2:1) | 13 (1.2:1) | 12 (2:1) | 10 (4:1) | 30 (2.3:1) | 147 (2.4:1) |
| Unaffected (F:M) | 28 (3:1) | 4 (3:1) | 64 (0.5:1) | 20 (1.2:1) | 51 (0.8:1) | 18 (1.6:1) | 73 (1.1:1) | 258 (1:1) |
| Spouse controls (F:M) |  |  | 2 (1:1) |  |  | 1 (0:1) | 1 (1:0) | 4 (1:1) |
| CIS | - | - | 2 (4%) | - | - | - | 2 (7%) | 4 (3%) |
| RR | 9 (41%) | 4 (80%) | 31 (56%) | 10 (77%) | 5 (42%) | 7 (70%) | 16 (53%) | 82 (56%) |
| SP | 1 (5%) | - | 12 (22%) | - | 5 (42%) | 1 (10%) | 10 (33%) | 29 (20%) |
| PR | 2 (9%) | - | - | - | 1 (8%) | - | - | 3 (2%) |
| PP | 3 (14%) | - | 1 (2%) | - | 1 (8%) | - | - | 5 (3%) |
| Unknown/Unclear | 7 (32%) | 1 (20%) | 9 (16%) | 3 (23%) | - | 2 (20%) | 2 (7%) | 24 (16%) |
| Onset Age | 35.1 [21-54] | 20.5 [18-23] | 30.1 [12-51] | 30.6 [19-49] | 29.7 [16-46] | 28.6 [16-47] | 30.2 [14-52] | 30.5 [12-54] |
| Disease duration | 11.1 [0-33] | 20.0 [8-32] | 14.3 [0-47] | 9.3 [0-25] | 12.8 [1-27] | 20.0 [2-41] | 15.1 [1-33] | 14.0 [0-47] |

*Cousins: at least one affected sample from one or more family branches, representing a fourth degree consanguinity.*

*Siblings+: two or more affected siblings, with or without other family members representing higher degrees of consanguinity.*

*Multi-generation, other: affecteds represent at least two generations, for example, proband and great-aunt.*

*Unknown/Unclear: participant is known to be affected, but MS sub-type information is not available.*
